# SensorTalk: An IoT Device Failure Detection and Calibration Mechanism for Smart Farming

**DOI:** 10.3390/s19214788

**Published:** 2019-11-04

**Authors:** Yi-Bing Lin, Yun-Wei Lin, Jiun-Yi Lin, Hui-Nien Hung

**Affiliations:** 1 Department of Computer Science, National Chiao Tung University, Hsinchu 300, Taiwan; liny@nctu.edu.tw (Y.-B.L.); iblis.dif01@nctu.edu.tw (J.-Y.L.); 2 College of Artificial Intelligence, National Chiao Tung University, Tainan 711, Taiwan; 3 Institute of Statistics, National Chiao Tung University, Hsinchu 300, Taiwan; hhung@stat.nctu.edu.tw

**Keywords:** failure detection, sensor calibration, smart farming

## Abstract

In an Internet of Things (IoT) system, it is essential that the data measured from the sensors are accurate so that the produced results are meaningful. For example, in AgriTalk, a smart farm platform for soil cultivation with a large number of sensors, the produced sensor data are used in several Artificial Intelligence (AI) models to provide precise farming for soil microbiome and fertility, disease regulation, irrigation regulation, and pest regulation. It is important that the sensor data are correctly used in AI modeling. Unfortunately, no sensor is perfect. Even for the sensors manufactured from the same factory, they may yield different readings. This paper proposes a solution called SensorTalk to automatically detect potential sensor failures and calibrate the aging sensors semi-automatically. Numerical examples are given to show the calibration tables for temperature and humidity sensors. When the sensors control the actuators, the SensorTalk solution can also detect whether a failure occurs within a detection delay. Both analytic and simulation models are proposed to appropriately select the detection delay so that, when a potential failure occurs, it is detected reasonably early without incurring too many false alarms. Specifically, our selection can limit the false detection probability to be less than 0.7%.

## 1. Introduction

The Internet of Things (IoT) connects sensors to actuators to collect and exchange data for various applications [1] such as smart grids [2], health care [3], unmanned aerial vehicles [4], narrowband IoT [5], and so on. Many IoT applications utilize the sensors to obtain data. These data are then served as inputs to, e.g., big data or artificial intelligence (AI) models to produce useful results through prediction. It is essential that the data measured from the sensors are accurate so that the results produced by the IoT applications are meaningful. Unfortunately, no sensor is perfect. Even for the sensors manufactured from the same factory, they may yield different readings. This is called manufacturing variation. Sensors may also be affected by the environment over time. That is, they become obsolete because they do not age well. In [6], a smart farm platform called AgriTalk was developed for soil cultivation with a large number of sensors. The produced sensor data were used in several AI models to provide precise farming for soil microbiome and fertility, disease regulation, irrigation regulation, and pest regulation [7]. In this application, it is important to identify sensor failure and to calibrate or replace the failed sensors in real time so that the data measured by the sensors are accurate.

Many schemes for sensor failure detection, identification, and accommodation (FDIA) have been proposed for aircraft systems, which assumed that the systems under test have many redundant sensors or analytical redundancy or are designed with expensive tools such as the X-Plane simulator [8]. In soil farming, the budget for IoT devices is limited, so the solutions for aircraft systems do not apply. In [9], the authors detected the failure of a sensor that measures surface temperature of an urban sewer. A predictive analytics solution that uses an autoregressive integrated moving average (ARIMA) model to incorporate a forecasting technique based on the past time series of sparse data was proposed. By assuming that forecasted and faulty data are Gaussian-distributed, the solution sets a criterion (i.e., 95% forecast interval and the continuity of faulty data) to detect anomalies and to issue a warning for sensor failure. In [10], a simple sensor fault detection algorithm was proposed, which utilizes multiple redundant sensors for failure detection. This solution is too expensive to deploy in the farm with a large number of sensors. This paper proposes a cost-effective sensor failure detection and calibration mechanism for farming applications. The scheme is called SensorTalk based on our belief that “*a sensor will tell you when it is sick and when it is dying, if you listen.*” All FDIA solutions (including SensorTalk) follow the sensor characteristics described in Section 2. The paper is organized as follows. Section 2 provides a tutorial for sensor accuracy and calibration. Section 3 describes sensors and actuators used in AgriTalk. Section 4 and Section 5 propose the SensorTalk approach for sensor failure detection and calibration.

## 2. Sensor Accuracy and Calibration

The most important performance index for a sensor is its accuracy, which is a combination of resolution and precision of that sensor [11]. Resolution specifies the smallest detectable incremental change of input signal that can be detected. That is, resolution is the ability to detect small changes in the measured signal. Precision is the ability to produce the same output for the same input. That is, to measure the same input several times, an ideal sensor outputs exactly the same value every time. Unfortunately, a real sensor outputs a range of values distributed in some manner relative to the actual value. Therefore, to have good precision, the discrepancy between the true value and the sensor’s mean value should be limited within a certain distance. Precision of a sensor is determined by noise and hysteresis. Every sensor is more or less affected by random noise. If signal-to-noise ratio is low, the sensor may not produce repeatable measurements. A sensor tends to read low with an increasing signal and high with a decreasing signal.

Note that both the noise and the hysteresis of a sensor cannot be compensated for [12]. However, if the sensor produces repeatable measurements with good resolution, then its accuracy can be enhanced through calibration. A sensor typically exhibits structural errors that make differences between the expected output and the measured output. Structural errors show up consistently whenever a new measurement is taken. Calibration removes structure errors of the sensor outputs and therefore improves the accuracy of the sensor. After being used for a period of time, the accuracy of a sensor may drop, and calibration is required.

Sensors need to be calibrated for accuracy on a regular basis [13]. In calibration, the characteristic curve is used to define a sensor’s response to an input. The calibration process maps the sensor’s response to an ideal linear response. In the ideal scenario, the characteristic curve will be a straight line. The greatest deviation of the characteristic curve from a reference line is described as non-linearity. The reference can be determined by, for example, limit point adjustment where the reference line runs through the start and end points of the characteristic curve. Calibration is achieved by the adjustment of the characteristic curve. Either single-point, two-point, or multiple-point adjustments can be performed. Single-point adjustment calibrates “offset”. When the sensor output is higher or lower than the ideal output, the difference between the actual output value and the measured output value is called the offset. If the characteristic curve had the same slope as the ideal one but crossed the Y-axis (output) at different points, then there is an offset. Two-point adjustment calibrates “sensitivity.” The slope of the characteristic curve is called the sensitivity of the sensor. In some sensors, the sensitivity is defined as the input parameter change required to produce a standardized output change. The sensor output changes at a different rate than the ideal rate, and this is known as the difference in the slope or the sensitivity error. Multiple-point adjustment calibrates “linearity.” A sensor is linear if its output is directly proportional to the input. The linearity of a sensor is expressed as the extent to which the actual measured curve of a sensor departs from the ideal curve. A real sensor may not have perfect linearity due to environmental factors such as temperature, humidity, vibration, and acoustic noise level. In fact, most sensors have non-linear characteristic curves. To achieve the best accuracy, the co-efficient values of the conversion function must be calibrated.

## 3. Farming Sensors and Actuators

**Figure 1:**
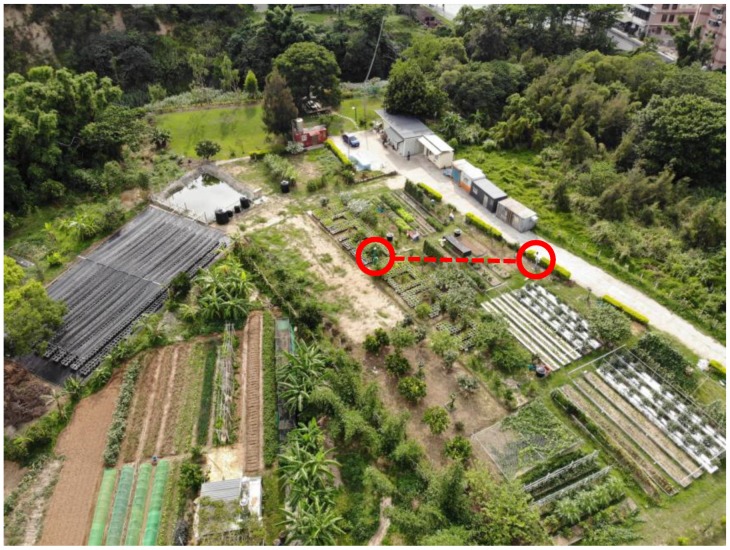
The Bao Farm (the weather stations are highlighted by the red circles).

AgriTalk smart soil farming system has been deployed in several farms in Taiwan [6]. This paper considers farms in three locations: both the Bao and the Longtan farms are located in low-altitude areas, and the Wufeng farm is located in a high-altitude area. Figure 1 shows the aerial photo of the Bao farm. AgriTalk deploys sensors of two categories. The sensors in the air are installed in a micro weather station, including those for CO_2_, temperature (−40 °C–65 °C), humidity (1–100% RH), barometric pressure, and ultraviolet (0~1800 W/m^2^). Each AgriTalk farm has installed two micro weather stations. In Figure 1, the weather stations are highlighted by the red circles. The dashed line in Figure 1 shows that two sensors of the same types are located some distance away in the farm.

**Figure 2:**
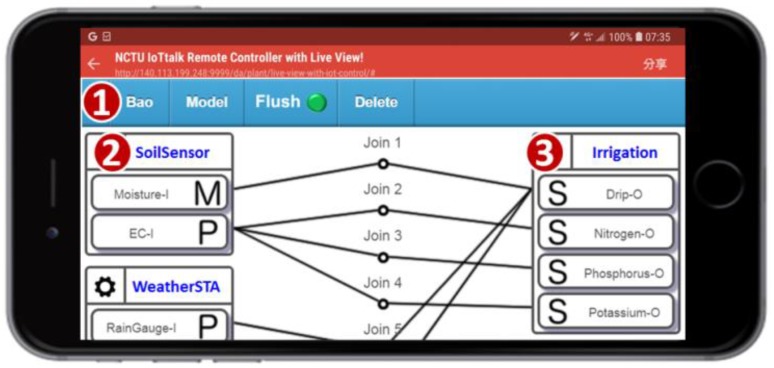
AgriTalk GUI for connecting IoT devices.

Another category of sensors is comprised of those inserted in the soil, and includes moisture, temperature, electrical conductivity (EC), and pH sensors. Multiple sensors of the same type are deployed in every farm area. Several actuators are used in AgriTalk, including the drippers, the repellent bulbs, the pest sprayers, and so on. The total number of deployed sensors and actuators are over 100 in the farm fields investigated in this paper. In AgriTalk, these sensors are connected to the actuators through a web-based, user-friendly graphical user interface (GUI). The AgriTalk GUI allows for the creation of smart farm tasks in a smartphone (Figure 2). These tasks are developed in several simple AgriTalk projects. In AgriTalk, the data generated from a sensor can be used in different projects, and an actuator can be controlled by multiple independent projects at the same time.

Consider a simplified “Irrigation” Project (Figure 2 (1)), where the drippers are controlled by the moisture sensors through the Join 1 connection in the GUI. The concept of a “Join connection” is described as follows. In the AgriTalk GUI, the soil sensors can be grouped and represented by an icon (called an input device; e.g., “SoilSensor” in Figure 2 (2)) placed at the left-hand side of the GUI window, and the irrigation actuators are grouped and represented by an icon (called an output device; e.g., “Irrigation” in Figure 2 (3)) placed at the right-hand side of the window. If an IoT device consists of both input and output parts, then it is represented by an input device icon and an output device icon (to be elaborated later). Inside an input device icon, there are one or more small icons that represent the sensors or the controls called input device features (IDFs). In Figure 2 (2), the SoilSensor input device has two IDFs Moisture-I and EC-I. Similarly, inside an output device icon, there are one or more small icons that represent the actuators called output device features (ODFs). In Figure 2 (3), the Irrigation output device has four ODFs: Drip-O, Nitrogen-O, Phosphorus-O, and Potassium-O.

The input and output device features interact through the Join lines, where each Join line has two segments connected by a circle. By clicking the circle, one can write a Python function for the connection. The following simple function at Join 1 triggers the dripper when the moisture is lower than 50%:

**if (float(moisture) < 50): return 1**

**else: return 0**

The above code implemented in Join 1 receives data from Moisture-I and stores them in the “moisture” variable. In the code, the returned value 1 means “turn on,” and the value 0 means “turn off.”

Through the Join links, AgriTalk manipulates the sensor data to precisely control the farm actuators. Clearly, correct sensor readings are essential for precision farming. Therefore, detecting sensor failure and calibrating aged sensors are the most important maintenance tasks for smart farm management. In [7], we showed that, by appropriately manipulating the accurate sensor data, the accuracy of predicting rice blast disease can be improved from 83 to 89%.

Sensor failures occur for different reasons. If the failure detected is caused by the sensor material defeat, then the sensor hardware must be replaced. On the other hand, if a failure occurs due to, e.g., sensor aging, then it can be calibrated and reused.

**Figure 3:**
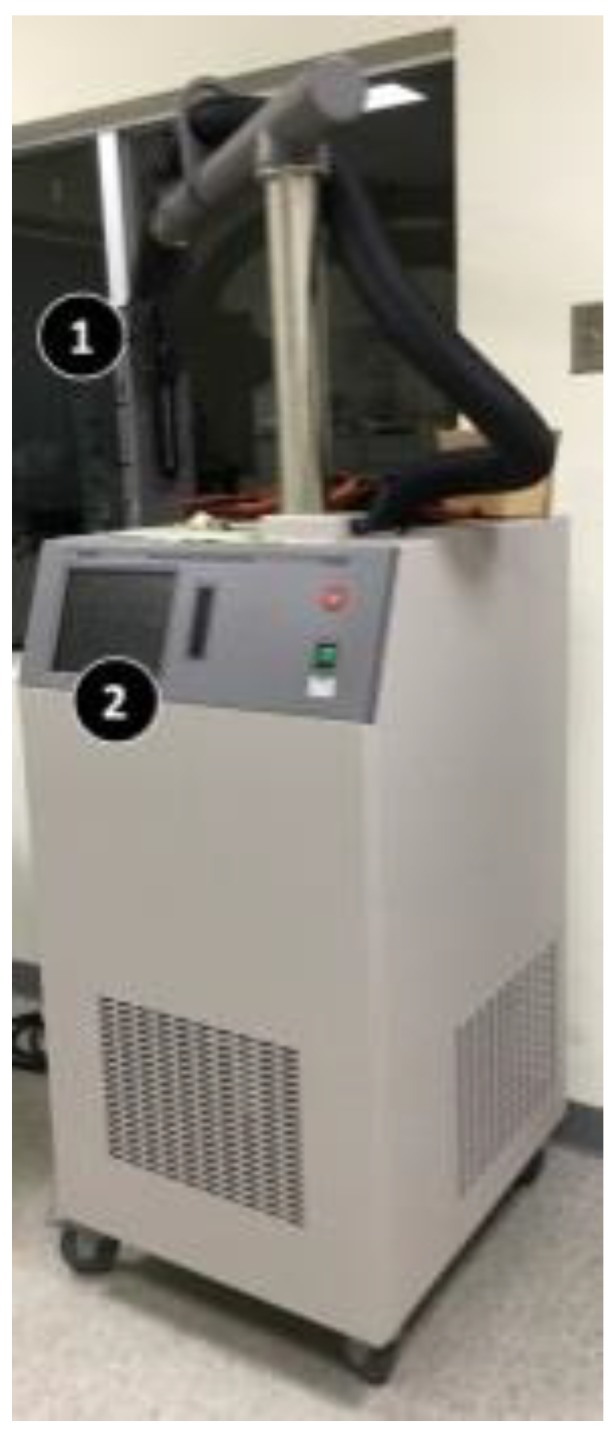
The Thermonics T-2500SE temperature forcing system.

There are many sensor calibration methods. To achieve the highest accuracy of calibration, the sensor (device) under test or DUT (device under test) is typically calibrated with a standard sensor (standard device, STD) under the controlled conditions, where the STD has been calibrated against NIST (National Institute of Standards and Technology) standards. The outputs of both sensors at the same conditions are grouped as a (DUT, STD) value pair to create a mapping table. This table is used to calibrate the characteristic curve of the sensor under test. The above procedure is typically performed in the laboratories to produce the accurate results. The most difficult part is to precisely create the various environment conditions to measure the sensor outputs. For example, the traditional temperature calibration is conducted in a laboratory using special equipment with advanced temperature testing capabilities such as the Thermonics T-2500SE (Thermonics Corp., Mansfield, USA) temperature forcing system [14]. T-2500SE uses a transparent tube (Figure 3 (1)) to place the temperature sensor to be calibrated. A technician sets a series of temperature values through a panel (Figure 3 (2)). Next, T-2500SE precisely changes the temperature in the tube following the preset temperature sequence. The outputs of the sensor under test (DUT) in the tube are sent to the reading meter of a laptop. The laptop then records the outputs from the temperature sensor, matches them with the actual (STD) temperatures controlled by T-2500SE, and finally generates a calibration table for the DUT sensor. Such a formal sensor calibration method is tedious and too luxurious for agriculture sensors operating in a large farm for two reasons. First, the calibration process cannot be automatically executed and regularly requires significant manual operations at the laboratory. Second, a sequence of environment conditions needs to be precisely generated (e.g., actual temperatures) for calibration at the laboratory, which means that special equipment is required. To resolve the above two issues, this paper proposes the SensorTalk mechanism to provide automatic sensor failure detection and semi-automatic calibration for AgriTalk. In our approach, the sensors are calibrated in the farms without the need of sending the sensors to the laboratories. The details are given in Section 5.

## 4. Failure Detection through Mutual Test

In soil cultivation, multiple sensors of the same type (e.g., moisture sensors) are deployed in a farm field. Therefore, two sensors of a same type placed within a short distance can automatically test each other. This process is called the mutual test for sensors of the same type or the “homogeneous” mutual test. Two highly related sensors of different types can also test each other. Similarly, a sensor and an actuator that are highly related can test each other (typically, the actuator is controlled by the sensor). Such process is called the “heterogeneous” mutual test. Based on the characteristics of the sensors and the actuators used in AgriTalk, the mutual test methods for failure detection are described in this section.

### 4.1. Homogeneous Mutual Test

**Figure 4:**
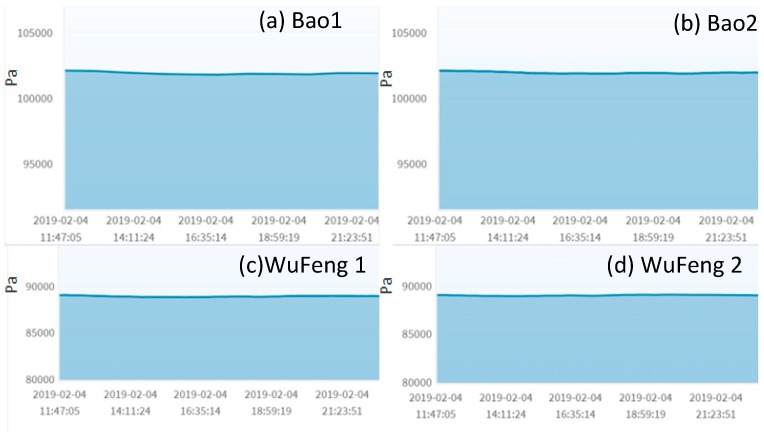
Barometric pressure.

The homogeneous mutual test involves two sensors of the same type, which are not located far away from each other. An example is the barometric pressure sensor that is most stable among all sensors used in AgriTalk. In Figure 4, the barometric pressure curves are almost flat. These output readings are affected by temperature, and the relationship between pressure and temperature is described by the Gay–Lussac Law. Due to air molecule collisions, this law says that, if the air temperature is increased, the pressure decreases. Different altitudes impact the temperature effect on air pressure due to disparities in air density. At a higher altitude, a higher temperature is required for the same air pressure as that at a lower altitude.

In Figure 4, the barometric pressure readings at Wufeng (in a high altitude mountain) are higher than that at the Bao field (at low altitude). An AgriTalk farm field typically deploys two micro weather stations at appropriate locations in the farm to report the weather conditions covering the whole farm field. If only one weather station is installed in the farm, we will use the nearby government weather station as the standard reference. Since the locations of the micro weather stations are at the same altitude, the barometric pressure readings are almost identical even if there is a distance of 10 m between them (see the (a)–(b) pair and the (c)–(d) pair in Figure 4).

**Figure 5:**
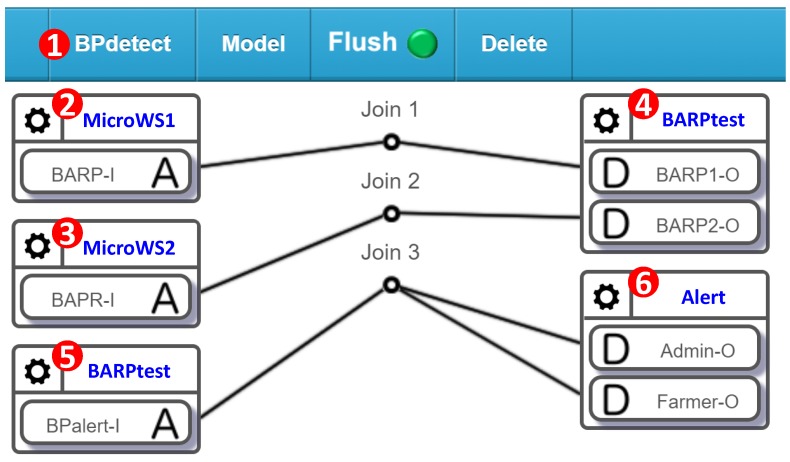
The mutual barometric pressure sensor test in the BPdetect project.

Therefore, these two barometric pressure sensors are highly related and can be mutually tested by an AgriTalk project “BPdetect” (barometric pressure detect; Figure 5 (1)) described as follows. The BPdetect configuration identifies the inconsistency of two barometric pressure sensors (BARP-Is) in the micro weather stations (Figure 5 (2) and (3)). This project develops a cyber IoT device called BARPtest (barometric pressure tester) with two ODFs and one IDF. BARP1-O and BARP2-O in the BPtest output device (Figure 5 (4)) receive the data from BARP1-I and BARP2-I, respectively, for a mutual test. The BARPtest device detects potential sensor failures in real time and outputs them through the BPalert-I IDF in its input device (Figure 5 (5)). This IDF is connected to the Admin-O and the Farmer-O ODFs of the Alert device (Figure 5 (6)). Admin-O then sends an alert message (through email, short message, or an announcement of a phone call) to the AgriTalk administer so that she/he can take action for sensor calibration described in the next section. The message is also sent to alert the farmer.

**Figure 6:**
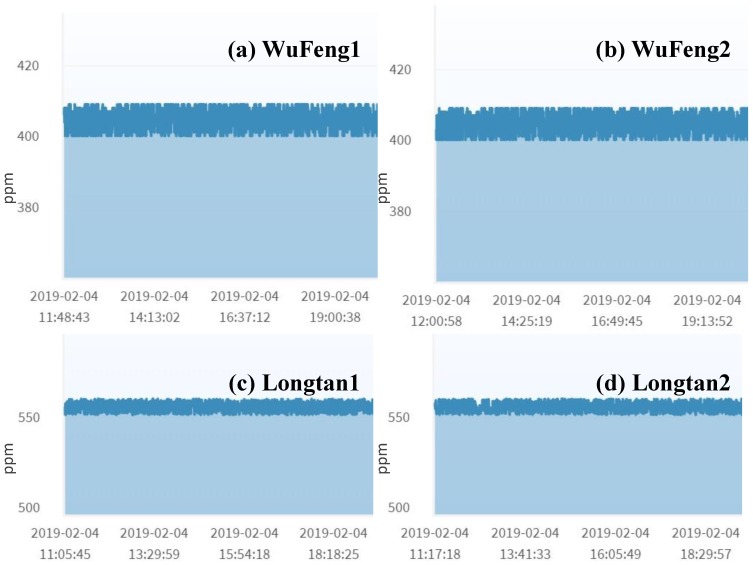
CO_2_ concentration.

Another good candidate for the homogeneous mutual test is the CO_2_ sensor. CO_2_ concentration in the air is affected by wind speed/direction, solar radiation, air temperature, rainfall, ultra-violet (UV) radiation, air pollution, and so on. Figure 6 shows that, although the CO_2_ concentration values produced by two sensors in the same area (Wufeng or Longtan) severely fluctuate, they are limited in small ranges with almost the same means. Therefore, these two sensors are good targets for the mutual test. If the CO_2_ concentrations are significantly affected by other factors (e.g., wind speed/direction), then the homogeneous mutual test with extra input factors described in Section 4.3 is conducted to better identify the failed sensor.

**Figure 7:**
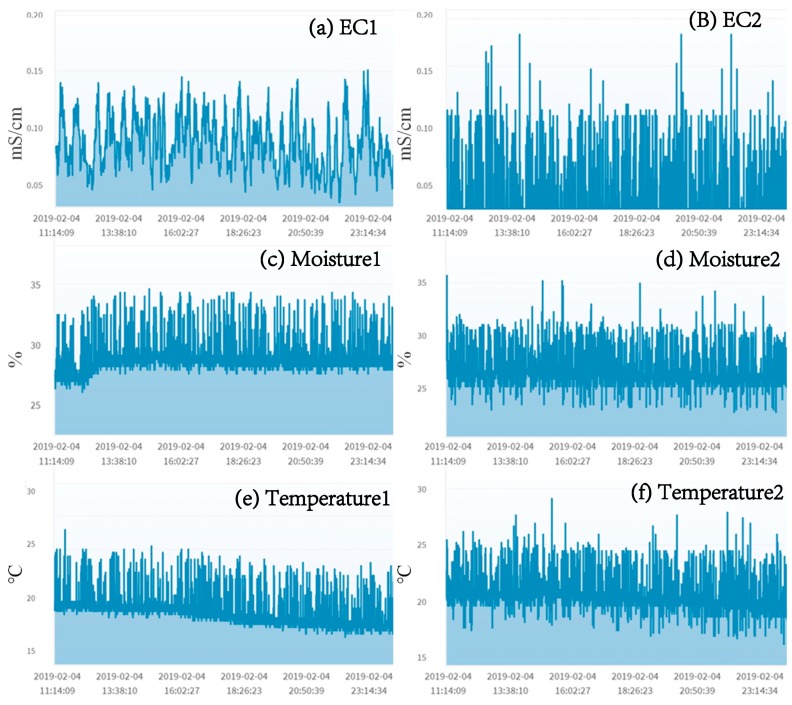
The soil sensors in nearby locations of the Longtan farm.

More difficult cases are those sensors inserted in the soils. The same types of soil sensors at nearby locations may give very different readings (Figure 7). However, if they show a relationship, they can still be tested (to be elaborated in Section 4.2 and Section 4.3).

**Figure 8:**
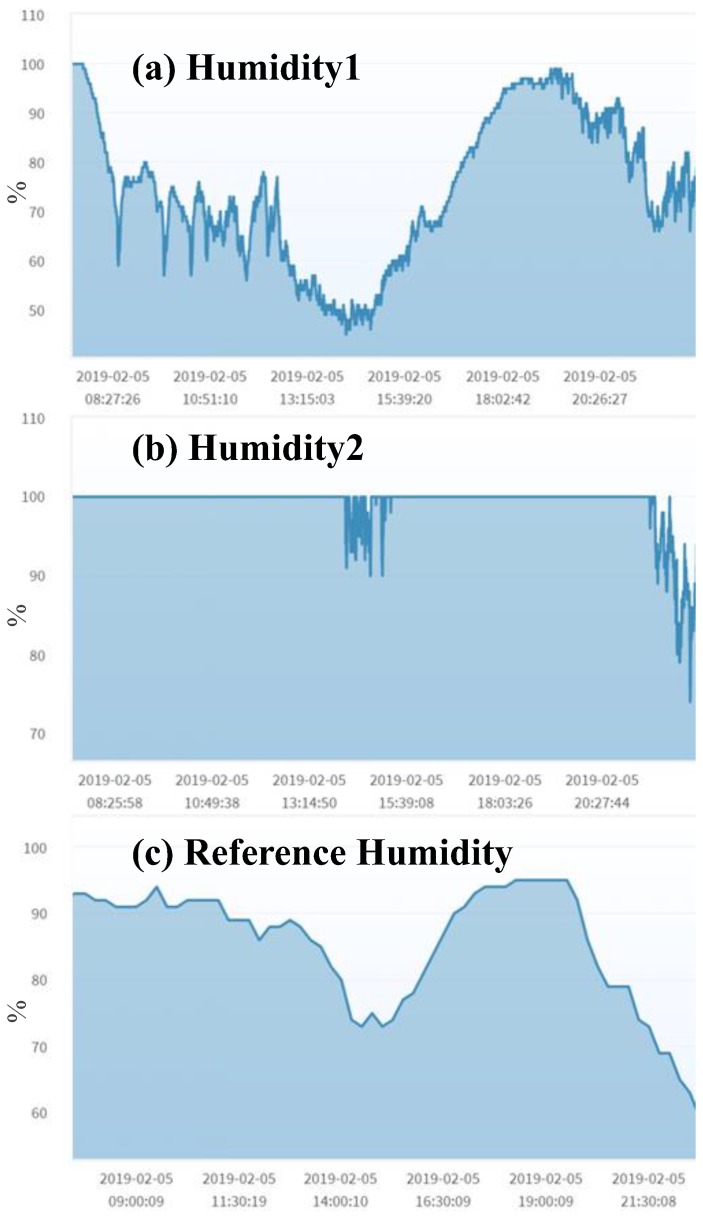
Humidity (Wufeng).

Note that, when the homogeneous mutual test shows a negative result, both sensors may fail as illustrated in Figure 8a,b. The correct readings of humidity are given in Figure 8c. In this case, both sensors should be calibrated.

### 4.2. The Heterogeneous Mutual Test

**Figure 9:**
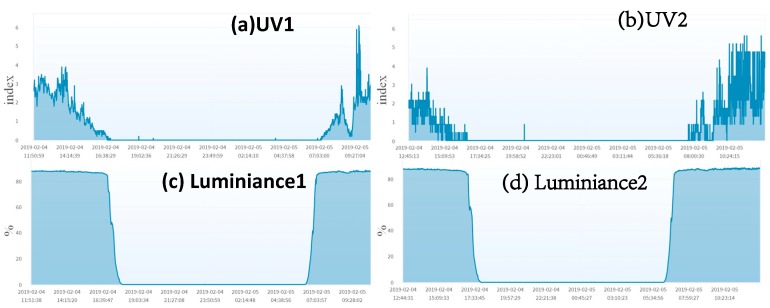
UV and Luminance (Wufeng).

The sensors of different types installed at the same location can be good candidates for the heterogeneous mutual test because they experience the same environment conditions. An example of the heterogeneous mutual test is for UV and luminance (they are installed in the same micro weather station). Figure 9 shows that a UV sensor and a luminance sensor at the same location are highly related. The AgriTalk project of failure detections for UV and luminance are the same as the BPdetect project, except that the IDFs are replaced by the UV and the luminance sensors. The details are omitted.

**Figure 10:**
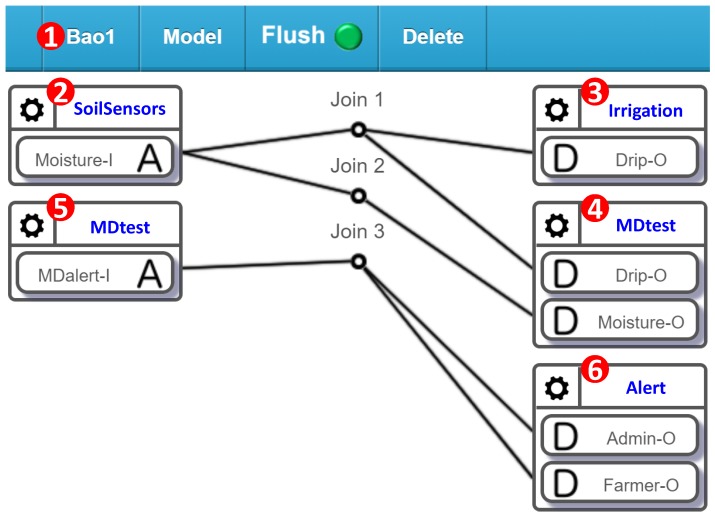
The Bao1 project for the heterogeneous mutual test.

In AgriTalk, every sensor affects at least one actuator. In [15] and the references therein, faults have been detected in the sensor and actuator interaction. If a sensor is used to control an actuator, then they are highly related and can be paired to mutually test each other. For example, in the AgriTalk irrigation system, the dripper is controlled by the moisture sensor as illustrated in Join 1 of Figure 2. Therefore, the moisture sensor and the dripper are highly related, and the heterogeneous mutual test can be performed by modifying the Bao project in Figure 2. The new project is called Bao1 (Figure 10 (1)), which is a subproject of the irrigation control system that only involves the moisture sensor (Moisture-I in Figure 10 (2)) and the dripper (Drip-O in Figure 10 (3)). We create a cyber test device “MDtest” (Moisture-Dripper tester) that has two ODFs and one IDF. In the output device (Figure 10 (4)), Drip-O receives the instruction sent from Join 1. Therefore, when it receives the value “1,” the MDtest knows that the dripper is turned on. When Drip-O receives the value “0,” the dripper is turned off. Moisture-O receives the measured moisture data through Join 2.

When the dripper is turned on, the MDtest starts monitoring the moisture readings until the dripper is turned off. If the irrigation system functions normally, then in a certain period of time $t_{T}$, the moisture should increase to reach a threshold value (e.g., 40% relative humidity). If the irrigation system does not work this way, then either the moisture sensor or the dripper actuator fails, and MDalert-I in Figure 10 (5) sends the alert message to the administrator and the farmer through Join 3. Note that the Alert device in Figure 10 (6) is the same cyber device in the BPdetect project (Figure 5 (6)). When both projects are activated, the administrator and the farmer will receive alerts from both projects if any IoT devices in these projects fail.

**Figure 11:**
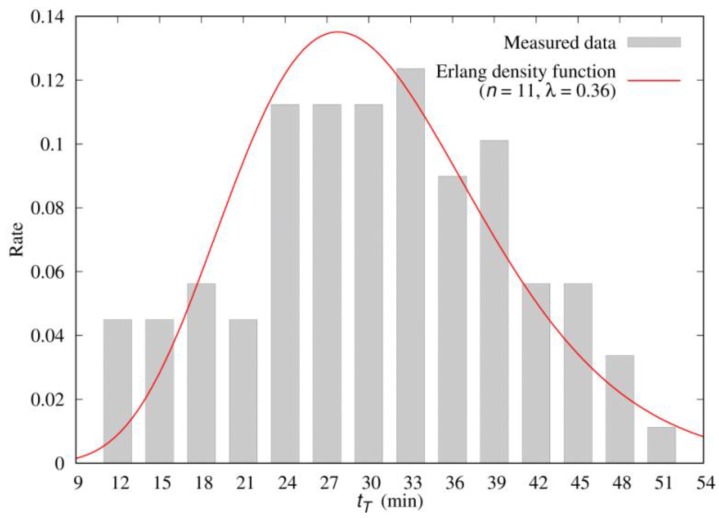
The histograms for *t_T._*

Note that $t_{T}$ is not a fixed value and can be appropriately described as a distribution. In Figure 10, MDtest accumulates the samples that are the periods between when Drip-O receives 1 and when it receives 0. The histogram of the measured data illustrated in Figure 11 indicates that the expected value E[*t_T_*] = 30.6 min, and the variance V[*t_T_*] = 0.094 E[*t_T_*]^2^ for the activation periods of Drip-O. In probability theory, the Erlang distribution is a two-parameter distribution. One is the scale parameter λ and the other is the shape parameter n. The shape parameter n can control the skewness of the distributions. From the histogram illustrated in Figure 11, the distribution of $t_{T}$ has a right skew; therefore, $t_{T}$ data are fit by an Erlang density function $f_{1}\left(t_{T}\right)$ with the shape parameter n and the scale parameter λ:

(1) $$f_{1}\left(t_{T}\right)=\frac{\lambda^{n}t_{T}{}^{n-1}e^{-\lambda t_{T}}}{\left(n-1\right)!}$$

where

n=11 and λ=0.36.

After estimating the parameters of the Erlang distribution, the Kolomogorov–Smirnov (K-S) test [16] is used to validate whether the distribution is actually an Erlang distribution.

If the moisture reading does not reach the threshold value (e.g., 40% in the case for Figure 11) in the period $t_{T}$, then we suspect that either the moisture sensor or the dripper actuator fails. It is clear that the actual $t_{T}$ value cannot be obtained, and we have to predict $t_{T}$ by another estimator τ. If the moisture reading does not reach the threshold value by the period τ, an alert message is sent to the administer to check if the sensor or the actuator fails. Period τ is called the detection delay. If τ is set too large, then the detection of failure is seriously delayed. On the other hand, if τ is set too small, then false alarms occur often (i.e., $\tau <t_{T}$ is likely to happen). In other words, when both the sensor and the actuator are normal, $\mathrm{Pr}[\tau <t_{T}]$ is the probability of false alarm. A detection delay τ should be selected such that τ is small enough to detect failure early and that false alarm does not frequently occur. Appendix A develops an analytic model to derive $\mathrm{Pr}[\tau <t_{T}]$, which indicates that it is appropriate to select a τ distribution with small variance and where $E\left[\tau \right]\geq 1.5E\left[t_{T}\right]$ so that false detection probability is less than 7%, and if we select $E\left[\tau \right]\geq 2E\left[t_{T}\right]$, the false detection probability is less than 0.7%.

The soil sensors are good targets for the heterogeneous mutual test because in AgriTalk, the soil sensors are integrated in one piece of hardware (the so-called three-in-one sensor device for EC, moisture, and temperature). In Figure 7a,c,e are placed in one location, and Figure 7b,d,f are in another. Based on the discussion in Section 4.1 and Section 4.2, we have the following fact.

**Fact 1.** A mutual test (either homogeneous or heterogeneous) of two IoT devices (two sensors or one sensor and one actuator) detects one of two situations. In Situation 1, the test shows the positive result that both IoT devices are normal. In Situation 2, the test shows the negative result that one or both IoT devices fail.

Situation 2 of Fact 1 cannot tell which device fails in one mutual test. Under certain conditions, we can identify the failed IoT device. Details are given in the next subsection.

### 4.3. The Combined Mutual Test

**Figure 12:**
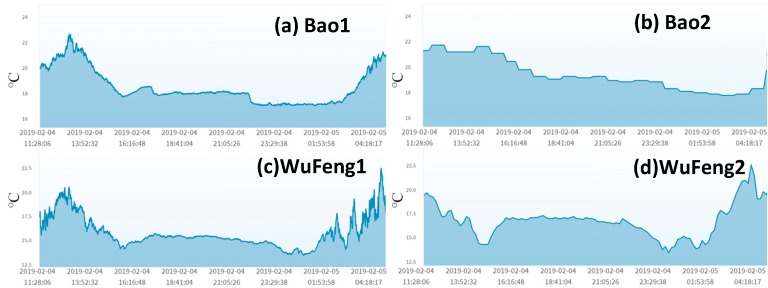
Air temperature.

Both homogeneous and heterogeneous mutual tests identify that the tested sensors may fail but cannot determine which of the two sensors fails. If heterogeneous and homogeneous mutual tests are combined, then it can be considered as a “homogeneous test with extra input factors.” In other words, in the combined mutual test, there are two same-type sensors under test, and different types of sensors or actuators can be used as extra input factors that assist in identifying which sensor fails. An example is the temperature sensor test in AgriTalk. Figure 12 shows the temperature readings of two micro weather stations in each of the Bao and the Wufeng fields. The figure indicates that the two reading curves in the same field are correlated. Therefore, the homogeneous mutual test applies to air temperature sensors. As we pointed out in Section 4.1, air temperature affects other air sensors, in particular, barometric pressure. If the readings of a barometric pressure sensor are correct, then they reflect the impact of the air temperature. In a mutual test of temperature, the temperature sensor with more accurate readings will relate better with the readings of the barometric pressure sensor. Therefore, an accurate barometric pressure sensor can be used in a mutual test to produce better results. In Figure 12, the temperatures in Wufeng are lower than that in Bao. At the same time, the barometric pressures in Wufeng are also lower than that in Bao as illustrated in Figure 4. Therefore, by conducting two homogeneous mutual tests and two heterogeneous mutual tests, there is a much better opportunity to identify the failed sensor.

**Figure 13:**
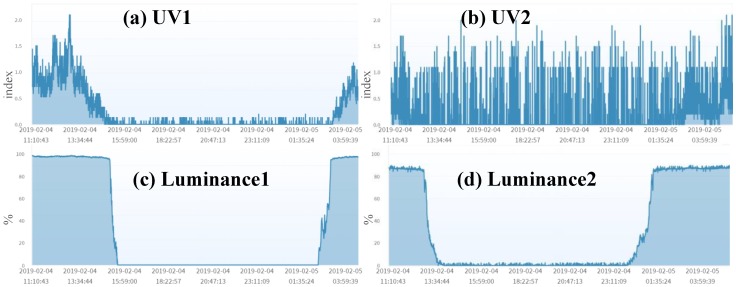
Luminance and UV (Longtan).

To further elaborate on the above statement, consider the UV and the luminance sensors in Longtan (Figure 13). It is fairly easy to identify that UV2 fails through the combined mutual test. When the homogeneous mutual test is conducted on the UV sensors, we find that they are not related correctly, and suspect that one of them may fail. When the homogeneous mutual test is conducted on the luminance sensors, we find that they are normal. Similarly, we conducted the heterogeneous mutual test on UV1 and Luminance1 to find that they are normal. We then conducted the heterogeneous mutual test on UV2 and Luminance2, and the result indicates that they are not related well. Based on the results of the four tests, we may conclude that Luminance1 is normal, UV1 and Luminance2 are likely to be normal, and UV2 is likely to fail. To identify that a sensor/actuator fails, we have the following fact:

**Fact 2.** A sensor/actuator $s_{1}$ fails if there exists a set S of sensors/actuators such that, for every $s_{2}\in S$, Situation 2 in Fact 1 occurs for the mutual test involving $s_{1}$ and $s_{2}$, and there exists $s_{3}\in S$ such that Situation 1 in Fact 1 occurs for a mutual test involving $s_{3}$ and another sensor/actuator.

Facts 1 and 2 assume that when a mutual test indicates a positive (negative) result, Situation 1 (2) holds. In some cases, the results may only guarantee that the situations are likely to hold with some probabilities. For example, although a mutual test indicates that both IoT devices are normal, an experienced IoT expert/farmer may still doubt that a device fails. Therefore, based on the expert’s experience, a probability may be given to an observed test result. Consider the example in Figure 13. Define the following events: A = {UV1 fails}, B = {UV2 fails}, C = {Luminance1 fails}, and D = {Luminance2 fails}. Denote $A^{c}$= {UV1 is normal} as the complementary event of A. Similarly, $B^{c}$, $C^{c}$, and $D^{c}$ are complementary events of B, C, and D, respectively. Let $\alpha_{1}$ be the probability (estimated by experienced experts) that the event A∪B occurs (Situation 2 of Fact 1), Similarly, $\alpha_{2}=\mathrm{Pr}\left[B\cup D\right],\alpha_{3}=\mathrm{Pr}\left[A\cup C\right]$, and $\alpha_{4}=\mathrm{Pr}\left[C\cup D\right]$. It is clear that $\mathrm{Pr}\left[A^{c}\cap C^{c}\right]=1-\alpha_{3}$ is the probability that Situation 1 of Fact 1 occurs. Therefore,

(2) $$B⊇B\cap \left(A^{c}\cap C^{c}\right)\;=\left[B\cap \left(A^{c}\cap C^{c}\right)\right]\cup \left[A\cap \left(A^{c}\cap C^{c}\right)\right]\;=\left(A\cup B\right)\cap \left(A^{c}\cap C^{c}\right).$$

Based on the above derivation,

(3) $$\mathrm{Pr}\left[B\right]\geq \mathrm{Pr}\left[\left(A\cup B\right)\cap \left(A^{c}\cap C^{c}\right)\right]\;=\mathrm{Pr}\left[A\cup B\right]\mathrm{Pr}\left[A^{c}\cap C^{c}|A\cup B\right]\;\geq \mathrm{Pr}\left[A\cup B\right]\mathrm{Pr}\left[A^{c}\cap C^{c}\right]=\alpha_{1}\left(1-\alpha_{3}\right).$$

Similarly,

(4) $$\mathrm{Pr}\left[B\right]\geq \mathrm{Pr}\left[\left(B\cup D\right)\cap (C^{c}\cap D^{c})\right]=\alpha_{2}\left(1-\alpha_{4}\right)$$

which results in

(5) $$\mathrm{Pr}\left[B\right]\geq \max\left[\alpha_{1}\left(1-\alpha_{3}\right),\;\alpha_{2}\left(1-\alpha_{4}\right)\right].$$

Suppose that $\alpha_{1},\alpha_{2}\to 1$. If $\alpha_{3},\alpha_{4}\to 1$, then we obtain the result Pr[B]≥0, which provides no information at all, and we cannot be sure that event B does happen. In other words, in these mutual tests, either $\alpha_{3}$ or $\alpha_{4}$ should approach 0 to provide useful information.

## 5. Calibration by Standard Sensors

**Figure 14:**
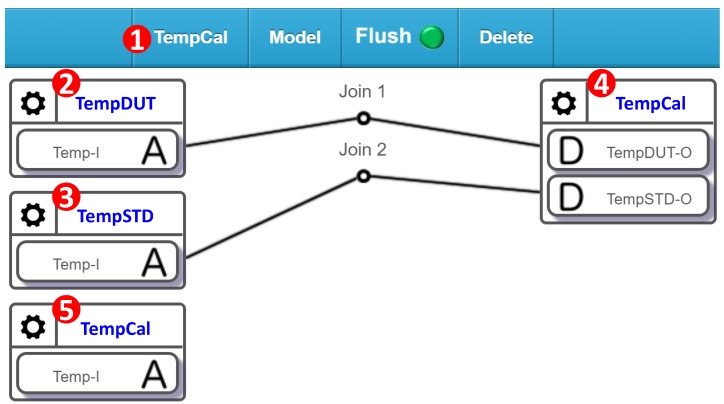
Temperature calibration.

After a failed sensor is found, either it is replaced or is calibrated. In the latter case, a standard sensor is used to build a mapping table to adjust the characteristic curve of the sensor under test. The sensor calibration in the laboratory is given in Section 3 (Lines 153–178). The beauty of SensorTalk is that it is able to perform the exact laboratory calibration procedure in the farm field. In AgriTalk, a failed sensor is manually replaced. The manual operation for sensor calibration is exactly the same as sensor replacement, except that we need to create a calibration project in the AgriTalk GUI (Figure 14 (1)). The TempDUT device (Figure 14 (2)) includes the temperature sensor to be calibrated (i.e., Temp-I) or the so-called “device under test” (DUT). AgriTalk provides a standard temperature sensor in the TempSTD device (Figure 14 (3)). Both the DUT sensor and the STD sensor are connected to the temperature calibration cyber device “TempCal.” When the paired (DUT, STD) values are sent to the input device of TempCal, they are saved as a mapping entry in the calibration table. The STD values are also sent out to control the AgriTalk actuators through Temp-I of the TempCal’s input device (Figure 14 (5)).

When the calibration process is complete, the standard sensor is unplugged, i.e., Join 2 is removed. TempCal will not receive new data from TempSTD-O, and whenever it receives a value from TempDUT-O, it maps this value to the calibrated value in the calibration table and sends out the mapped value through Temp-I of TempCal. Note that the mappings are created based on the calibration process (for offset, sensitivity, or linearity) described in Section 2.

**Figure 15:**
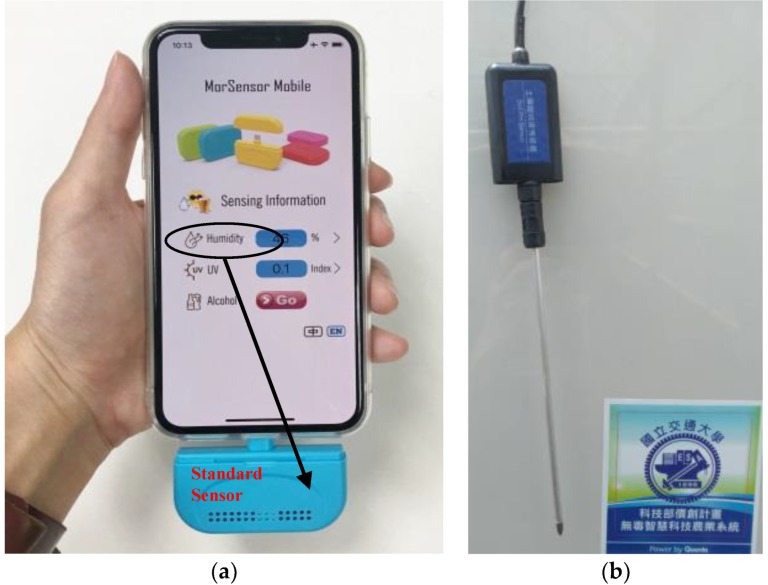

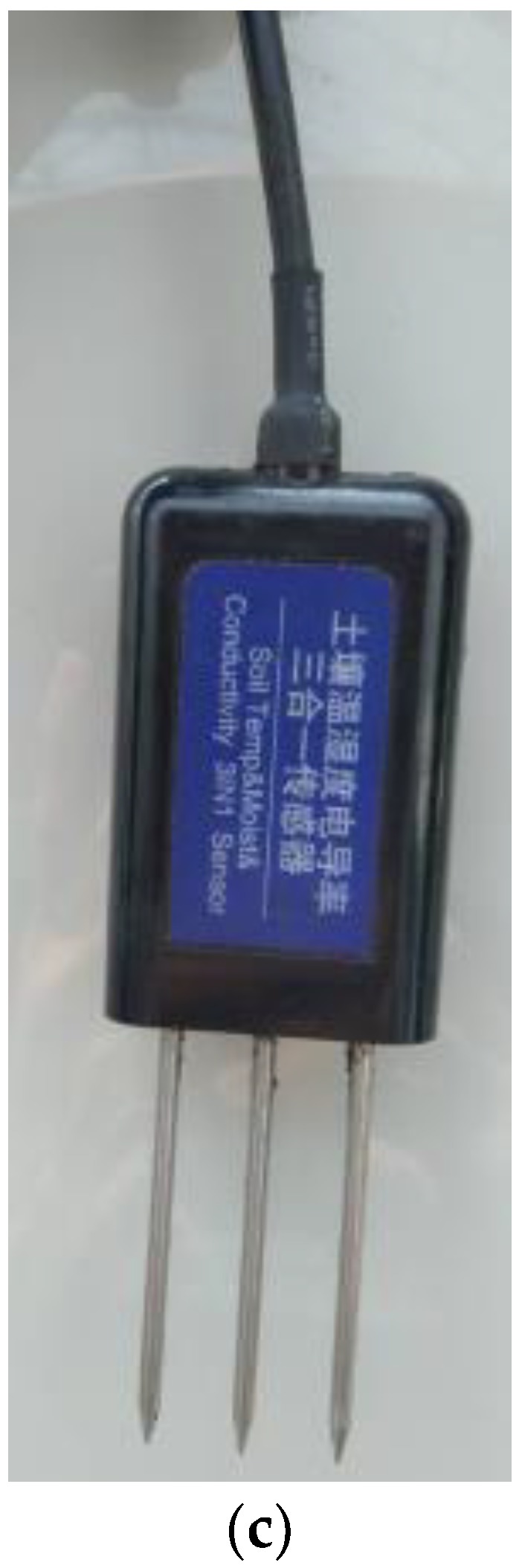
Smartphone-based portable STD value generator; (**a**) STD sensors in the air; (**b**) and (**c**) STD sensors in the soil.

AgriTalk’s sensor calibration is conducted in the farm by placing the DUT and the STD sensors at the same location so that they experience the same environment conditions. In this way, we avoid sending the DUT sensors to the laboratories for expensive and tedious calibrations. In the laboratory, we can fully control the whole range of the environment conditions to build the calibration table. In the farm, the weather will automatically generate the environment conditions for calibrating the sensors. In AgriTalk, we collect all data sent from a sensor and dynamically find the maximum and the minimum values of the collected data. This approach is called dynamic ranging [17]. These two values define the operation range of a sensor. Therefore, before an AgriTalk calibration is complete, the STD sensor should output a sufficiently large number of test points that include at least three values: the threshold value (e.g., when a moisture sensor reports 50% of relative humidity, AgriTalk will activate the dripper), the max value, and the min value. To perform calibration in the farm field, we have developed a smartphone-based STD value generator [18], where a mobile app is installed to a smartphone to connect to the AgriTalk server (e.g., the TempSTD icon in Figure 14 (3)), and the standard sensors are connected to the smartphone through a type-C/USB connection. Figure 15a illustrates the configuration for the standard air sensors, and Figure 15b,c illustrates the standard soil sensors connected to the smartphone through the type-C/USB line. This portable equipment can be easily carried and used in the farm field to generate the calibration table. Table 1 and Table 2 show the calibration tables for a temperature sensor and a humidity sensor in the Bao farm, respectively.

The sensor calibration table is automatically generated by SensorTalk (Figure 15) to record both the outputs of STD and the DUT under the same environmental conditions in the farm field. The outputs of the DUT are then compared with those of the STD, and the adjusted (STD) results are saved in a calibration table. For example, in the Bao farm, if the TempDUT device (Figure 14 (2)) outputs a temperature value of 26.52 °C, the TempCal device (Figure 14(5)) will output the adjusted temperature value of 24.98 °C according to Table 1.

## 6. Conclusions

This paper proposes SensorTalk, which automatically detects sensor and actuator failures and semi-automatically calibrates aging sensors. The AgriTalk smart farm platform for soil cultivation was used as an example to show how failures can be detected in a large number of sensors. Mutual tests were designed for detecting failures of farming sensors and actuators. We showed how multiple mutual tests can be performed to identify failed sensors/actuators. A cost-effective method to conduct sensor calibration in a farm field was also proposed. Examples of temperature and humidity calibration tables were presented. Within a detection delay, the SensorTalk solution can detect a failure when the sensors control the actuators. Both analytic analysis and simulation experiments were conducted to appropriately select the detection delay so that, when a potential failure occurs, it is detected reasonably early without incurring too many false alarms. Our study indicates that it is appropriate to select a τ distribution with small variance and $E\left[\tau \right]\geq 2E\left[t_{T}\right]$ so that false detection probability is less than 0.7%.

As a final remark, the AgriTalk farming sensor based on SensorTalk has won the CES’s 2020 Innovation Awards Showcase held at the Commercial Electronic Show (CES) at Las Vegas due to two major contributions. First, the calibration operation at a farm (instead of at a laboratory) results in a feasible and low-cost method. Second, the novel analytic analysis for detection delay determines an appropriate threshold for practical failure detection.

## Figures and Tables

**Table 1:** Calibration mappings for a temperature sensor in the Bao farm.

DUT (°C)	26.52	27.41	28.90	31.45	32.73	33.00	32.73	33.79	34.67	35.33	35.90	35.88
STD (°C)	24.98	25.79	27.19	29.68	29.85	31.22	31.61	32.35	33.82	33.86	34.48	34.51

**Table 2:** Calibration mappings for a humidity sensor in the Bao farm.

DUT (%)	78.47	77.31	72.41	63.63	57.47	58.28	50.80	51.06	46.56	45.86	45.07	43.71
STD (%)	77.24	76.56	72.29	64.31	58.80	58.67	52.58	52.16	48.85	47.69	46.92	46.09

**Figure A1:**
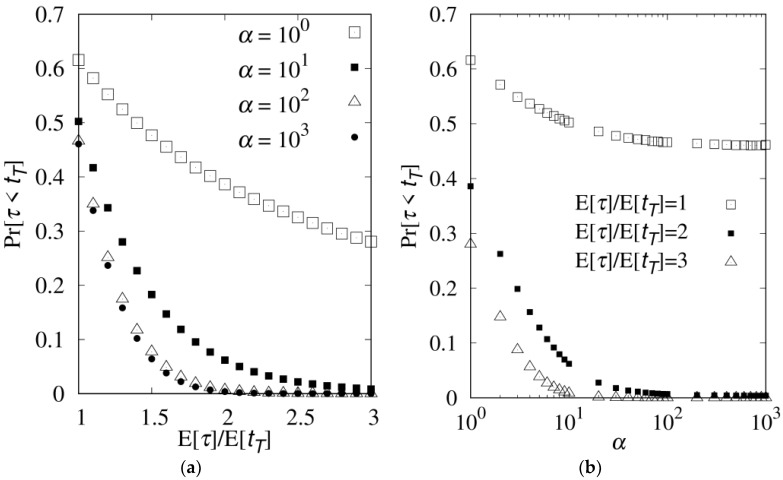
$\mathrm{Pr}\left[\tau <t_{T}\right]$ against E[τ] (**a**) and α (**b**).

## Appendix A. False Alarm Probability and Detection Delay

This appendix uses both analytic and simulation models to evaluate the false alarm probability and uses these models to select an appropriate τ value such that the delay is balanced against the false alarm probability.

In general, the estimator τ can be a random variable with an arbitrary density function $f_{2}\left(\tau \right)$ (practically, fixed τ is often selected). From Equation (1), the false alarm probability $\mathrm{Pr}[\tau <t_{T}]$ is

(A1) $$\mathrm{Pr}\left[\tau <t_{T}\right]=\int_{\tau =0}^{\infty }f_{2}\left(\tau \right)\int_{t_{T}=\tau }^{\infty }f_{1}\left(t_{T}\right)dt_{T}d\tau \;=\int_{\tau =0}^{\infty }f_{2}\left(\tau \right)\left[\overset{n-1}{\underset{i=0}{\sum }}\left(\frac{\lambda^{i}t_{T}{}^{i}e^{-\lambda \tau }}{i!}\right)\right]d\tau \;=\overset{n-1}{\underset{i=0}{\sum }}\int_{\tau =0}^{\infty }f_{2}\left(\tau \right)\left(\frac{\lambda^{i}\tau^{i}e^{-\lambda \tau }}{i!}\right)d\tau \;=\overset{n-1}{\underset{i=0}{\sum }}\left(\frac{\lambda^{i}}{i!}\right)\int_{\tau =0}^{\infty }\tau^{i}f_{2}\left(\tau \right)e^{-\lambda \tau }d\tau$$

Let $f_{2}^{*}\left(s\right)$ be the Laplace of $f_{2}\left(\tau \right)$. Since

(A2) $$\int_{\tau =0}^{\infty }\tau^{i}f_{2}\left(\tau \right)e^{-s\tau }d\tau ={\left(-1\right)}^{i}\left[\frac{f_{2}^{*\left(i\right)}\left(s\right)}{ds^{i}}\right]$$

Equation (A1) is re-written as

(A3) $$\mathrm{Pr}\left[\tau <t_{T}\right]=\overset{n-1}{\underset{i=0}{\sum }}{\left(-1\right)}^{i}\left(\frac{\lambda^{i}}{i!}\right){\left[\frac{f_{2}^{*\left(i\right)}\left(s\right)}{ds^{i}}\right]⎤}_{s=\lambda }$$

Without loss of generality, assume that τ is a Gamma random variable with the shape parameter α and the scale parameter β. The Laplace transform $f_{2}{}^{*}\left(s\right)$ is then expressed as

(A4) $$f_{2}{}^{*}\left(s\right)=\frac{\beta^{\alpha }}{{\left(s+\beta \right)}^{\alpha }}$$

The Gamma distribution is a general form of the Erlang distribution and is widely used in telecommunications network modeling [19]. With Gamma τ, Equation (A3) is re-written as

(A5) $$\mathrm{Pr}\left[\tau <t_{T}\right]=\overset{n-1}{\underset{i=0}{\sum }}\left(\begin{array}{c} \alpha +i-1\\ i \end{array}\right)\left[\frac{\lambda^{i}\beta^{\alpha }}{{\left(\lambda +\beta \right)}^{\alpha +i}}\right]$$

Let

(A6) $$x=\frac{\lambda }{\lambda +\beta }\;\mathrm{and}\;X\left(\alpha \right)=\overset{n-1}{\underset{i=0}{\sum }}\left(\begin{array}{c} \alpha +i-1\\ i \end{array}\right)x^{i}$$

Therefore, from Equations (A5) and (A6), we have

(A7) $$\mathrm{Pr}\left[\tau <t_{T}\right]={\left(1-x\right)}^{\alpha }X\left(\alpha \right)$$

Let

(A8) $$Y_{j}=\overset{n-1}{\underset{i=0}{\sum }}i^{j}x^{i}.$$

In Equation (A6), if α=1, then

(A9) $$X\left(1\right)=Y_{1}=1+x+x^{2}+x^{3}+\ldots +x^{n-1}=\frac{1-x^{n}}{1-x}$$

Substitute Equation (A9) into Equation (A7) to yield

(A10) $$\mathrm{Pr}\left[\tau <t_{T}\right]={\left(1-x\right)}^{\alpha -1}\left(1-x^{n}\right)\;={\left(\frac{\beta }{\lambda +\beta }\right)}^{\alpha -1}\left[1-{\left(\frac{\lambda }{\lambda +\beta }\right)}^{n}\right]$$

In Equation (A6), if α=2, then from Equation (A9),

(A11) $$X\left(2\right)=\overset{n-1}{\underset{i=0}{\sum }}\left(i+1\right)x^{i}=\overset{n-1}{\underset{i=0}{\sum }}ix^{i}+Y_{1}$$

In (A11), we have

(A12) $$\overset{n-1}{\underset{i=0}{\sum }}ix^{i}=Y_{2}=x+2x^{2}+\ldots +\left(n-1\right)x^{n-1}$$

Multiply *x* on both sides of the equation to yield

(A13) $$xY_{2}=x^{2}+2x^{3}+\ldots +\left(n-1\right)x^{n}$$

and

(A14) $$\left(1-x\right)Y_{2}=Y_{1}-1-\left(n-1\right)x^{n}$$

After rearrangement, we obtain

(A15) $$Y_{2}=\frac{Y_{1}-1-\left(n-1\right)x^{n}}{1-x}=\frac{Y_{1}-nx^{n}}{1-x}-Y_{1}$$

Substitute Equation (A15) into Equation (A11) to yield

(A16) $$X\left(2\right)=\frac{X\left(1\right)-nx^{n}}{1-x}=\frac{1-\left(n+1\right)x^{n}+nx^{n+1}}{{\left(1-x\right)}^{2}}$$

Substitute Equation (A16) into Equation (A7), and we obtain

(A17) $$\mathrm{Pr}\left[\tau <t_{T}\right]={\left(1-x\right)}^{\alpha }\left[\frac{1-\left(n+1\right)x^{n}+nx^{n+1}}{{\left(1-x\right)}^{2}}\right]\;={\left(\frac{\beta }{\lambda +\beta }\right)}^{\alpha -2}\left\{1-\left[\frac{\lambda +\left(n+1\right)\beta }{\lambda +\beta }\right]{\left(\frac{\lambda }{\lambda +\beta }\right)}^{n}\right\}$$

In Equation (A6), if α=3, then

(A18) $$X\left(3\right)=\overset{n-1}{\underset{i=0}{\sum }}\frac{\left(i+2\right)!x^{i}}{2!i!}=\overset{n-1}{\underset{i=0}{\sum }}\left(\frac{i^{2}+3i+2}{2}\right)x^{i}$$

Substitute Equations (A9) and (A15) into Equation (A18), we have

(A19) $$X\left(3\right)=\frac{Y_{3}+3Y_{2}}{2}+Y_{1}$$

where

(A20) $$Y_{3}=\overset{n-1}{\underset{i=0}{\sum }}i^{2}x^{i}\;=\frac{2Y_{2}-Y_{1}+1-n^{2}x^{n}+2nx^{n}-x^{n}}{1-x}\;=\frac{2Y_{2}-n\left(n-1\right)x^{n}}{1-x}-Y_{2}$$

Substitute Equation (A20) into Equation (A19) to yield

(A21) $$X\left(3\right)=\frac{Y_{3}+Y_{2}}{2}+Y_{2}+Y_{1}$$

By using Equations (1), (A15), (A20), and (A21), we obtain $\mathrm{Pr}\left[\tau <t_{T}\right]$ through Equation (A7).

A simulation model has been developed to compute $\mathrm{Pr}\left[\tau <t_{T}\right]$. The implementation of the simulation is similar to the one in [20]. Details are omitted. We use Equations (A10) and (A17) to validate the simulation model with various α,β,λ, and n values, and the discrepancies between the simulation and the analytic results are within 1%. Figure A1 plots $\mathrm{Pr}\left[\tau <t_{T}\right]$ for various α,β values with n=11 and λ=0.36 (the parameters obtained from Figure 11). The figure indicates that $\mathrm{Pr}\left[\tau <t_{T}\right]$ reduces as α increases (the variance become small). It is trivial that $\mathrm{Pr}\left[\tau <t_{T}\right]$ increases as E[τ] increases. In our example, it is appropriate to select α≥0 and $E\left[\tau \right]\geq 1.5E\left[t_{T}\right]$ so that false detection probability is less than 7%, and if $E\left[\tau \right]\geq 2E\left[t_{T}\right]$ is selected, the false detection probability is less than 0.7%.

## References

[1] Ni J., Zhang K., Lin X., Shen X. (2018). Fog Computing for Internet of Things Applications: Challenges and Solutions. IEEE Commun. Surveys Tutor..

[2] Chiu T.-C., Shih Y.-Y., Pang A.-C., Pai C.-W. (2017). Optimized Day-Ahead Pricing with Renewable Energy Demand-Side Management for Smart Grids. IEEE Internet Things J..

[3] Shih Y.-Y., Hsiu P.-C., Pang A.-C. (2019). A Data Parasitizing Scheme for Effective Health Monitoring in Wireless Body Area Networks. IEEE Trans. Mobile Comput..

[4] Sharma V., Srinivasan K., Chao H.-C., Hua K.-L., Cheng W.-H. (2017). Intelligent deployment of UAVs in 5G heterogeneous communication environment for improved coverage. J. Netw. Comput. Appl..

[5] Huang C.-W., Tseng S.-C., Lin P., Kawamoto Y. (2019). Radio Resource Scheduling for Narrowband Internet of Things Systems: A Performance Study. IEEE Netw..

[6] Chen W.-L., Lin Y.-B., Lin Y.-W., Chen R., Liao J.-K., Ng F.-L., Chan Y.-Y., Liu Y.-C., Wang C.-C., Chiu C.-H. (2019). AgriTalk: IoT for Precision Soil Farming of Turmeric Cultivation. IEEE Internet Things J..

[7] Chen W.-L., Lin Y.-B., Ng F.-L., Liu C.-Y., Lin Y.-W. (2019). RiceTalk: Rice Blast Detection using Internet of Things and Artificial Intelligence Technologies. IEEE Internet Things J..

[8] Hussain S., Mokhtar M., Howe J.M. (2014). Sensor Failure Detection, Identification, and Accommodation Using Fully Connected Cascade Neural Network. IEEE Trans. Ind. Electron..

[9] Thiyagarajan K., Kodagoda S., Nguyen L.V. Predictive Analytics for Detecting Sensor Failure Using Autoregressive Integrated Moving Average Model. Proceedings of the 12th IEEE Conference on Industrial Electronics and Applications (ICIEA).

[10] Oh W. A Simple Sensor Fault Detection Algorithm. Proceedings of the Int’l Conference on Computer Science, Data Mining & Mechanical Engg. (ICCDMME’2015).

[11] Gajda J., Sroka R., Stencel M., Zeglen T., Piwowar P., Burnos P., Marszalek Z. Design and accuracy assessment of the multi-sensor weigh-in-motion system. Proceedings of the IEEE Instrumentation and Measurement Technology Conference.

[12] Jerath K., Brennan S., Lagoa C. (2018). Bridging the gap between sensor noise modeling and sensor characterization. Measurement.

[13] Oh H.S., Kim U., Kang G., Seo J.K., Cho H.R. (2018). Multi-Axial Force/Torque Sensor Calibration Method Based on Deep-Learning. IEEE Sens. J..

[14] Advanced Test Equipment Rentals. https://www.atecorp.com/products/thermonics/t-2500se.

[15] Nguyen T.-D., Chiem T.-P., Duonga T.-H., Le D.-H., Nguyen X.-A. (2017). On improving agricultural IoT management process for fault detection. J. Inf. Telecommun..

[16] Sachs L. Critical Values for the Kolmogorov-Smirnov Test for Goodness of Fit. https://web.archive.org/web/20180219145741/http://www.mathematik.uni-kl.de/~schwaar/Exercises/Tabellen/table_kolmogorov.pdf.

[17] Lin Y.-B., Lin Y.-W., Huang C.-M., Chih C.-Y., Lin P. (2017). IoTtalk: A Management Platform for Reconfigurable Sensor Devices. IEEE Internet Things J..

[18] Lin Y.-B., Huang C.-M., Chen L.-K., Sung G.-N., Yang C.-C. (2018). MorSocket: An Expandable IoT-based Smart Socket System. IEEE Access..

[19] Yang S.-R., Lin P., Huang P.-T. (2008). Modeling Power Saving for GAN and UMTS Interworking. IEEE Trans. Wirel. Commun..

[20] Lin Y.-B., Tseng H.-C. (2019). FishTalk: An IoT-based Mini Aquarium System. IEEE Access..

